# From Constellation Dithering to NOMA Multiple Access: Security in Wireless Systems

**DOI:** 10.3390/s21082752

**Published:** 2021-04-13

**Authors:** Krystian Grzesiak, Zbigniew Piotrowski

**Affiliations:** Institute of Communications Systems, Faculty of Electronics, Military University of Technology, 00-908 Warsaw, Poland

**Keywords:** steganography, covert channel, physical layer security, low probability of detection

## Abstract

In recent years, there has been a noticeable increase in interest in the possibilities of embedding additional data in the constellation of an already existing information signal in radio technology. This solution more precisely is based on adding a low power signal (or signals) to a stronger signal (cover). As will be described in the article, this technique is used in numerous radio communication areas, such as watermarking, covert channel creation, and multiple access techniques. Typically, those areas are considered as independent research topics. Our comparison suggests that these areas are closely related. In this article, a comprehensive survey of the implementation of signal superposition is conducted with an emphasis on the similarities and differences between individual solutions. Since the nature of the signal model entails certain problems in the security area, we provide the reader with a review of the state-of-the-art research on this topic, including the PLS (physical layer security) and LPD (low probability of detection) issues.

## 1. Introduction

**Figure 1:**
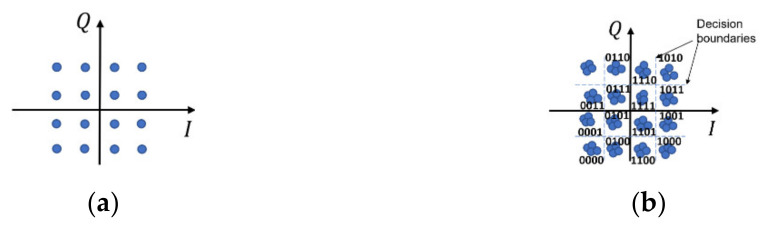
A 16-QAM signal constellation: (**a**) ideal; (**b**) affected by disturbances.

Nowadays, digital modulations are widely used in wireless communication systems. A modulated signal can be depicted by a constellation diagram. Each point of the constellation represents a symbol of transmitted information (one or more bits). In the transmitter, the constellation mapper is responsible for mapping the bits. On the receiving side, the demapper assigns the symbol to a given point of the constellation. Figure 1a shows an ideal constellation for a 16-QAM signal. This constellation is symmetric around the X and Y axes and all points are equally spaced. Different signal distortions modify the ideal constellation. In the output of the transmitter, the constellation is typically inaccurate, for example, due to thermal noise and hardware imperfections. The signal in the channel is subject to many disturbing influences, including attenuation, fading, and noise. Hence, Figure 1b corresponds more to real conditions, and demodulation in the receiver is made based on decision areas.

The EVM (error vector magnitude) is a commonly used metric of the quality of a signal. EVM measures the difference between the measured signal and an ideal reference signal. Different standards specify the acceptable maximum values of EVM. For example, according to [1], the maximum EVM for the transmitter in the case of a 16-QAM modulation scheme equals 12.5%. Obviously, any distortions in the constellations are undesirable. However, there has been a concept that by using a high-quality transmitter (and receiver) it is possible to send additional information as a permissible error. The transmitted signal is created on the basis of the superposition of two (or more in the general case) digital signals of different power. Apart from the implementation details, in the literature, such solutions fall within the following issues: “dirty modulation”, “constellation dithering”, “shaping constellation” and, as a consequence of application extensions, as P-NOMA (power-domain non-orthogonal multiple access) multiple access systems. Thus, we are dealing with the implementation of the same technique but for different applications, and therefore we are dealing with different requirements for the additional transmitted information.

In our article, we analyze the typical applications and scenarios where the superposition of signals was adopted. We start with watermarking as the simplest implementation, which is the basis for understanding more complex applications like covert channels and multiple access systems. As will be shown, the main advantage of the analyzed method of signal embedding is simplicity (transmitter and receiver) and quite good performance. The simplicity of the receiver can be understood as a post-IC (interference cancellation), where interference constitutes the stronger (strongest) of the signals. This method represents the most effective IC-based reception technique in terms of bit-error-rate (BER) performance [2]. 

The most interesting aspect of the communication (covert and multiple users) is security issues. Though the main advantage of the presented method is simplicity, the research to ensure security (in a sense low probability of detection or physical layer security) still seems to be developed.

Our survey is different from existing surveys and books. This article attempts to provide a comprehensive survey of the existing applications of the superposition of signals. So far, watermarking, covert channel, and NOMA were considered as independent issues. Our goal was to find a relationship between these techniques. There are potential opportunities in this approach. We are convinced that it is worth considering applying methods typical for the covert channel in order to increase the security of NOMA system. This should be carried out in our future research.

Besides the method described in this topic, there is an alternate manner of embedding information. At the very least, DPC (dirty paper coding) should be mentioned [3,4,5], which can yield the optimal performance but with a quite high implementation complexity. However, since this paper deals with a specific solution, the DPC techniques will not be discussed further.

## 2. Superposition of Signals as a Watermarking

Watermarking [6] is associated primarily as a method of securing multimedia copyrights (e.g., image, video, audio, and text). By securing copyrights, additional information can be placed on carriers to be authenticated, and the media distribution tracked. A far as multimedia are concerned, an essential requirement for the watermarking method is to ensure that the processed signal does not change its quality upon reception. 

Watermarking may also apply to a radio link. It is applied when we want to ensure additional security of the system through the scheme authentication [7]. Unlike multimedia watermarking, the physical layer’s watermarking quality is not stable as the radio channel easily degrades it. Watermarking of the physical layer can be a covert modulation on the cover (carrier) signal [8,9], the greatest value being that it does not require any additional signal bandwidth. A watermarking scheme for an OFDM signal [9] includes the term constellation dithering (CD). We can also find similar solutions in [10,11]. Another solution, not undertaken in this paper, is constellation shifting, proposed by Tan et al. [12] and analyzed by Jiang et al. [13]. A comparison of the methods mentioned above has been included in the study [14].

The following simplified model can represent the operation of applying a watermark to the radio signal, as described in References [9,10,11]. For a Tx transmitting a digital information signal $x_{1}$, watermark $x_{2}$ is applied. The information signal (carrier) and the watermark are allocated power $P_{1}$ and $P_{2}$ (let us assume $P_{1}>P_{2}$), respectively, so that the condition is satisfied where $P=P_{1}+P_{2}$. The signal is therefore transmitted in the form

(1) $$s\left(t\right)=\sqrt{P_{1}}x_{1}\left(t\right)+\sqrt{P_{2}}x_{2}\left(t\right).$$

Due to the effect of additive noise $n\left(t\right)$ and channel gain h, a signal is received in the form

(2) $$y\left(t\right)=h\times s\left(t\right)+n\left(t\right)$$

To receive a watermark $\hat{x_{2}\left(t\right)}$, the carrier’s signal must be deducted from the received signal. Primary points of the carrier constellation (without channel distortions) $x_{1demap}$ are determined in the so-called “demapper”:

(3) $$\hat{x_{2}\left(t\right)}=x_{2}\left(t\right)+n\left(t\right)=\frac{1}{\sqrt{P_{2}}}\left(\frac{y\left(t\right)}{h}-\sqrt{P_{1}}x_{1_{demap}}\left(t\right)\right)$$

**Figure 2:**
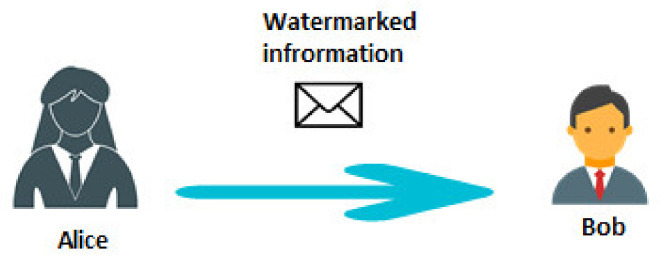
Watermarking of a radio signal. The sender sends the watermarked information to the recipient.

The radio signal watermarking system model can be represented by the model (Figure 2) where Alice sends the watermarked information to Bob.

We have one sender and one recipient of both information (cover) and watermarking in the presented model. The information of both is subject to the same radio channel distortion. The watermarking process affects the transmitted cover signal. Assuming ideal demapper performance, the SNR (SINR) values for the cover ($SINR_{1}$) and the watermark ($SNR_{2}$) are, respectively,

(4) $$SINR_{1}=\frac{{\left|h\right|}^{2}P_{1}}{{\left|h\right|}^{2}P_{2}+\sigma_{N}}$$

and

(5) $$SNR_{2}=\frac{{\left|h\right|}^{2}P_{2}}{\sigma_{N}},$$

where $\sigma_{N}$ is the noise variance $n\left(t\right)$ with spectral density $N\left(f\right)$.

Channel capacity R is the sum of cover $R_{1}$ and watermark capacity $R_{2}$ ($R=R_{1}+R_{2}$):

(6) $$R_{1}=Wlog_{2}\left(1+SINR_{1}\right),$$

where as for the watermark,

(7) $$R_{2}=Wlog_{2}\left(1+SNR_{2}\right)$$

It should be noted that usually during watermarking, the amount of information $x_{2}$ (in terms of the number of bits per watermark) is not an essential element; it can be assumed that the choice of power $P_{2}$ is only intended to guarantee the possibility of receiving the information by the recipient.

## 3. Superposition of Signals as a Covert Channel

The first to use the term “covert channels” was Lampson [15]. He focused on the exchange of data between programs and defined the covert channel as a communication channel that is not at all designed and intended for the transmission of information. It is currently assumed that any method of communication used to illegally transmit information, which violates the system security policy, is a covert channel. Data security is indemnified at the flow control level between the sender and the authorized recipient of the data. All of that is being done so that the data do not leak beyond the established connection. Firewalls control the connection for digital lines. However, under favorable conditions, security can be bypassed using covert channels. In the network application layer, it is possible to create a covert channel by transmitting information hidden in the text, image, and in the lower layers using network protocols and timing. Information is carried in reserved bits at the network layer or by changing the timing (timing channels). Firewalls usually catch the other methods. 

Contrary to the network layer, the signal’s physical layer provides further possibilities to hide the transmitted information. In general, the implementation of covert communication in the radio channel compared to wired communication has its advantages and disadvantages [16]. 

In wired communications, it must be ensured that the channel is not distorted by network devices on either side of the covert channel. In wireless communication, the range between two points is limited by the transmitter’s power and the parameters of the receiver, e.g., the sensitivity of signal reception. In wireless communication, we deal with noise, interference and drop-outs that can seriously degrade transmission capabilities.

In the literature, we can find several ways to create covert channels based on the physical layer of the radio signal. Hijaz and Frost [17] proposes to allocate unused subcarriers and the so-called “virtual subcarriers” to maintain adequate inter-channel spacing. The whole range of solutions for creating a covert channel is included in Classen et al. [18]. Based on the OFDM signal, Classen et al. [18] propose the use of training sequences by applying covert information to them in the form of PSK modulation, applying the covert information in the form of a shift of the signal carrier frequency, using similar to Hijaz and Frost [17] virtual subcarriers and placing changes in the cyclic prefix. The disadvantage of all these [18] solutions is the low bit rates obtained.

**Figure 3:**
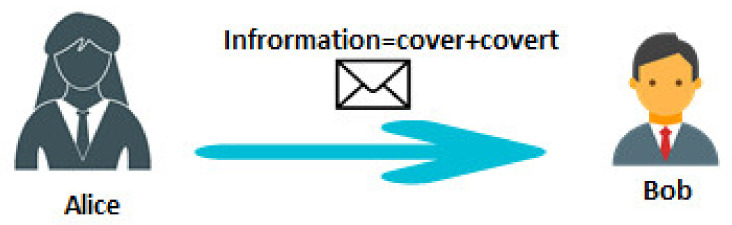
A covert channel.

The technique based on placing information on the constellation of the signal seems to be a better solution than watermarking. Dutta et al. [19] describes the embedding of points in a constellation offset from the original points and adopts the term “dirty constellation”. A similar solution is proposed by Cao et al. [20], although it focuses more on covert channel detection methods. Creating a covert channel by superimposing a multivalent amplitude-phase modulation on a carrier constellation was proposed by D’Oro et al. [21]. In its concept, this idea is no different than in [19,20]. The information transmission model for a covert channel may be presented as follows (Figure 3).

As in watermarking, the system describing the covert channel consists of the sender and the recipient, but in this case, the information carrier plays the role of hiding the exchanged covert information. The patterns on SNR, SINR and the capacity of the channel R remain the same as for the watermarking.

It should be noted that the definition of a covert channel does not specify the conditions that define the required degree of transmitted information concealment, nor the criteria or method of its determination. On the other hand, however, we would like to know under what conditions an outsider would detect the covert signal. The analysis for the detection of a covert channel leads to the simple conclusion that for an observer, Steve, who is closer to the sender (radio transmitter) and thus with lower channel attenuation (gain $h_{2}>h_{1}$), it will be easier to detect the existence of a covert channel. On the other hand, the closer-located Steve needs less transmitted signal power. Another interpretation of the Alice–Bob–Steve model is that the Alice–Bob signal is deliberately jamming to conceal the Alice–Steve transmission.

**Figure 4:**
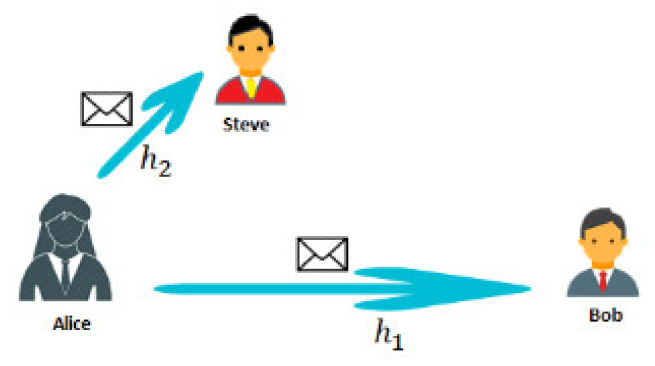
Separation of covert channel information for a cover and the covert information.

According to Figure 4, Steve and Bob use the same resources (frequency) simultaneously. Therefore, they share resources, which is especially desirable in modern radio systems. So far, in the models considered, only the superposition of two signals has been taken into account (in watermarking and covert channels). However, this concept can be extended to embed other signals—superposition of many signals, carrying information for various users with different channel gains. Thus, adapting the same method of collective signal creation, we obtain the multiple access technique, which appears in the literature under the name P-NOMA.

## 4. Superposition of Signals as a Multi-Access Systems NOMA

By watermarking and covert channels, we move to the P-NOMA multiple access techniques, which are combined by the same signal but from different applications. As shown in the following chapters, the methods of analysis in terms of transmission security also remain common. Of course, in multi-access techniques, the most critical issue is to guarantee connectivity between users. NOMA first appeared in Benjebboour et al. [22], which then triggered an avalanche of work on this technique. The name NOMA comes from the fact that the previously existing multiple access techniques (TDMA—time division multiple access; FDMA—frequency division multiple access; and CDMA—code division multiple access) ensured mutual non-interference of the signals of individual users. In NOMA, the individual users’ signals interfere with the others (inter-user interference), and hence the transmission is non-orthogonal. The NOMA receiver operates similarly to the previously described watermarking and covert channel systems, except that, theoretically, it anticipates an unlimited number of users. The method of extracting the stream of individual users (previously described as the use of a demapper) is known as SIC (successive interference cancellation), which is to reflect the gradual separation of signals from individual users, leading to a gradual reduction in interference. Compared to orthogonal multiple access, NOMA does not allow for full interference elimination; hence, this solution is a compromise between the obtained bit rate and the quality of the connection.

There are generally two types of NOMA systems. The first one is classified as the power-domain NOMA (P-NOMA, the subject of this article) and the second is the code-domain NOMA (C-NOMA). In C-NOMA, the users are assigned spreading code strings, with the difference, however, that they are not orthogonal (more users/strings than the number of orthogonal strings for a given spreading sequence would result).

The P-NOMA, multiple access description, corresponds to a covert channel shown in Figure 4 enhanced with multiple users in mind. There are also definitions specific to multi-access systems; hence, the following will present a description of the P-NOMA system in its simplest form [23], which assumes a single base station (BS) and two users equipped with a single antenna. Thus, the signal is not operated with the term cover and covert information compared to the covert channel description. Assume that $x_{1}$ and $x_{2}$ are signals transmitted by the BS to User 1 and User 2, respectively. The signal transmitted by the BS is an overlay (superposition) of the signals:

(8) $$s=\overset{2}{\underset{i=1}{\sum }}\sqrt{P_{i}}x_{i}$$

where $P_{i}$, i= 1, 2 constitutes the power transmitted to User i, and $x_{i}$ a unit-power message signal, i.e., $E\left\{{\left|x_{i}\right|}^{2}\right\}=1$ whereas $E\left\{\cdot \right\}$ denotes the expected value. The total power of Users u1 and u2 equals P ($P=P_{1}+P_{2}$). In practice, the value P is predefined and distributed as $P_{1}$ and $P_{2}$ according to the adopted scheme of power allocation (PA). The signal at the i user receiver may be written as

(9) $$y_{i}=h_{i}s+n_{i},$$

where $h_{i}$ is the channel gain (channel ratio) and the $n_{i}$ noise of the variance $\sigma_{N}$. Noise includes interferences and, in the case of multicellular systems, the inter-cellular interference.

According to the SIC method, the user channels (signals) are decoded sequentially, starting from the weakest user channel (lowest value ${\left|h_{i}\right|}^{2}/\sigma_{N}$) up to the strongest (highest value ${\left|h_{i}\right|}^{2}/\sigma_{N}$). According to this order, each user can eliminate interference from other users that are decoded after. Therefore, the user u2 (with the maximum value ${\left|h_{1}\right|}^{2}/\sigma_{N}$ channel strength), also called the strong user, can eliminate the interference from the weak (with lower value ${\left|h_{i}\right|}^{2}/\sigma_{N}$) user u1. The user signal strengths and the decoding order are determined by the BS based on the CSI (channel state information). To reduce the SINR, the weaker user is allocated more power compared to the stronger user. For both users, assuming that ${\left|h_{1}\right|}^{2}/\sigma_{N}<{\left|h_{2}\right|}^{2}/\sigma_{N}$ (thus, $P_{1}>P_{2}$) only the other user performs the SIC elimination. First, it decodes signal $x_{1}$ of User 1 and then deducts it from the received signal $y_{2}$ to ultimately decode its own signal. User 1 treats $x_{2}$, the User 2 signal, as interference; therefore, it decodes its signal from $y_{1}$ without executing SIC.

If the interference elimination procedure is perfect, the achievable transmission speed $R_{i}^{NOMA}$ in the NOMA system for the “i” user in the W=1 Hz band is

(10) $$R_{1}^{NOMA}=log_{2}\left(1+\frac{P_{1}{\left|h_{1}\right|}^{2}}{P_{2}{\left|h_{2}\right|}^{2}+\sigma_{N}}\right),\;$$

(11) $$R_{2}^{NOMA}=log_{2}\left(1+\frac{P_{2}{\left|h_{2}\right|}^{2}}{\sigma_{N}}\right)$$

Summation channel throughput $R^{NOMA}=R_{1}^{NOMA}+R_{2}^{NOMA}$. The equations suggest that the BS controls each user’s transmission rate by choosing a power allocation coefficient $\alpha_{1}$ and $\alpha_{2}$ with the values of $\alpha_{1}=\frac{P_{1}}{P}$ and $\alpha_{2}=\frac{P_{2}}{P}$. To compare NOMA multi-access with those traditionally used in systems, e.g., LTE with the orthogonal multiple access (OMA) system, let us assume that two users share the 1 Hz bandwidth. It means that User 1 applies the $W_{1}$ Hz bandwidth, and User 2 applies the rest of the bandwidth $W_{2}=1-W_{2}$. The signal strength ratios for individual users should remain the same as for the NOMA ($\alpha_{1}:\alpha_{2}=P_{1}:\;P_{2}$).

In this case, the achievable speed rate for the OMA user $R^{OMA}$ may be recorded as

(12) $$R_{1}^{OMA}=W_{1}log_{2}\left(1+\frac{P_{1}{\left|h_{1}\right|}^{2}}{\sigma_{N}}\right),$$

$$R_{2}^{OMA}=W_{2}log_{2}\left(1+\frac{P_{2}{\left|h_{2}\right|}^{2}}{\sigma_{N}}\right).\;$$

Summation channel throughput equals $R^{OMA}=R_{1}^{OMA}+R_{2}^{OMA}$. The equations suggest that there is no interference between the users as there was in the NOMA system.

**Figure 5:**
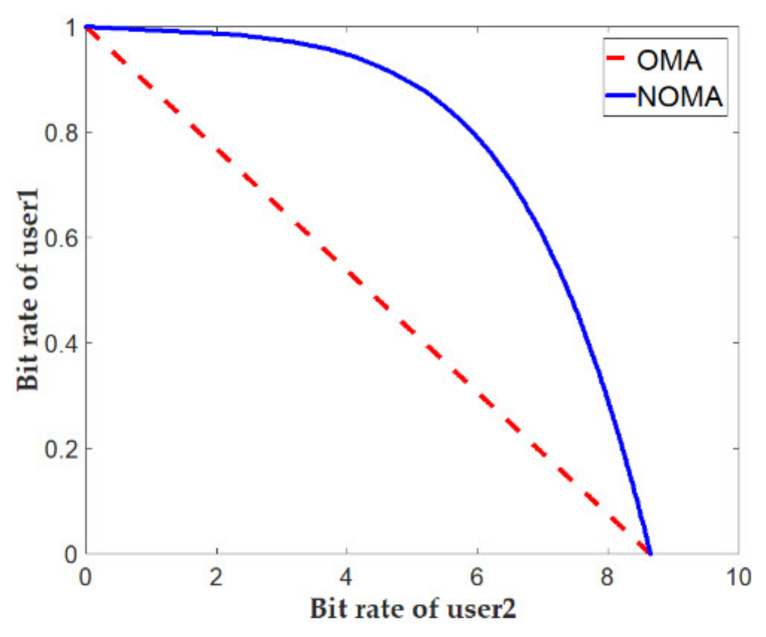
Examples of transmission rate changes for the analyzed cases of the orthogonal multiple access (OMA) and non-orthogonal multiple access (NOMA) systems.

Figure 5 shows the relationships between the achieved transmission rates for the OMA and NOMA systems. It was assumed that $h_{1}=\sqrt{10}$ whereas $h_{2}=0.2\times h_{1}$ and p  = 40. With OMA systems, increasing the speed means increasing the bandwidth for one user at the expense of the other. In NOMA systems, the transmission speed is more closely related to the users’ power mutual relation. However, it should be noted that the benefits of the NOMA systems result from the various assumed parameters $h_{i}$. When the channel parameters and noise power values for each user are the same ($h_{1}=h_{2}$, $N_{f,1}=N_{f,2}$), the NOMA and OMA systems would produce similar results.

The NOMA signal could be considered a covert channel where the weak user is not informed of other users’ existence. Thus, without performing the SIC process, it would not know about the transmission to another user (we assume that the user does not perform steganalysis). Its signal would be a cover for covert information. A user who does not perform the SIC process (and at the same time has the lowest channel gain) can be called non-NOMA.

## 5. Security Aspects of Watermarking, Covert Channels and NOMA

NOMA’s multi-access systems are not intended to hide transmissions, but the system’s physical security constitutes a question. In the case of a covert channel, we would like to know under what boundary conditions the existence of this channel can be detected. Similar considerations may apply to watermarking. As it was shown, the idea of watermarking, covert channel creation and NOMA have common elements; therefore, their analysis may be similar.

The system security analysis can be carried out for two conditions: physical layer security (PLS) and low probability of detection (LPD). The first of these conditions applies mainly to multi-access systems. The second one applies mainly to watermarking and covert channels. The differences between LPD and PLS are most easily presented by employing typical problem analysis models for these areas of science [24].

**Figure 6:**
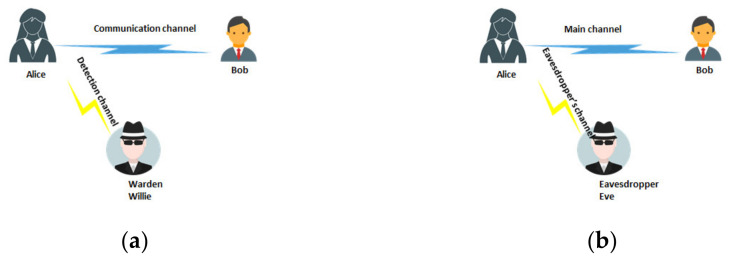
Difference between low probability of detection (LPD) (**a**) and physical layer security (PLS) (**b**).

According to Figure 6, one can notice that the main difference between LPD and PLS is the intruder’s purpose. The LPD scheme is based on the classic basic model introduced to science by Simmons [25] and defining the prisoner’s problem. Alice wants to send the information to Bob so that Willy the warden overlooks the fact of transmission. The warden’s job is to detect communication between Alice and Bob (and not to receive data). The Alice–Bob channel is detected through the Alice–warden channel. In the case of PLS, we are dealing with a wire-tap, the purpose of which is to learn the content of the information transmitted between Alice and Bob [26,27,28,29]. Both the Alice–Bob and Alice–Eve channels are used for communication. In a mathematical sense, LPD is an issue related to binary detection (whether or not there is a transmission). PLS is a matter of ensuring the security of the physical layer in connectivity.

In both models, it is necessary to assume two types of intruders [30]: external and internal. As an internal intruder, we mean one of the system’s legal users, while an external intruder remains outside the group of legitimate system users. In the analysis of covert channels and watermarking, the terms of the internal and external intruder are in place, and the terms informed and uninformed receiver are usually used interchangeably. An extension of the intruder concept is also the intruder/passive and active receiver. In the case of the latter, communication may be impaired.

### 5.1. PLS

Physical layer security protects the content of the information being sent [26,27,28,29]. Often, PLS (physical layer security) is considered a field complementary to cryptography [30] and in some cases even as a substitute for it. PLS uses the changing characteristics of radio communication, including channel randomness, drop-outs, interference and noise, to prevent eavesdropping (information retrieval). Compared to traditional cryptographic methods, PLS does not deal with the cryptographic protection of the transmitted information. By definition, PLS serves to achieve secrecy data transmission. In the literature, we can find notions like perfect secrecy or weak secrecy [31]. The basis of the secrecy systems was developed by Shannon [32]. Later, the basics of covert transmission were defined by Wyner in the SISOSE system (single-input single-output single-antenna eavesdropper). He proved that information could be sent securely if the channel between the transmitter and the intruder’s receiver (eavesdropper) is a distorted version of the primary information transmission channel, i.e., between the transmitter and the legitimate receiver [33]. Shannon defines perfect secrecy when the observation offers no information to the adversary, which means that the adversary’s channel capacity equals zero. Wyner replaced Shannon’s perfect secrecy with the weak secrecy condition, namely, the asymptotic rate of the leaked information should vanish as the code length tends to infinity.

Shannon and Wyner derived the theoretical limits on the secrecy of the systems. In other words, they follow the information-theoretic principles that generally deal with how much one can secure the communication, rather than how to do it [34]. Thus, to properly evaluate secrecy performance there are certain metrics used. Most common are SINR-based metrics such as secrecy capacity, secrecy outage probability, and intercept probability [28,35].

Secrecy capacity is the most used metric in PLS evaluation.$\;C_{s}$ is defined as the capacity difference between the main and wiretap channels; it defines the maximum secret rate at which the secret message reliably recovers at the legitimate user while remaining unrecoverable at the eavesdropper:

(13) $$C_{s}=\max\left\{C_{B}-C_{E},\;0\right\}$$

where $C_{B}$ and $C_{B}$ are capacities of the main and wiretap (eavesdropper) channels.

This metric is later extended to outage secrecy and outage secrecy rate probability [36].

Secrecy outage probability is defined as the probability that the secrecy capacity falls below a target secrecy rate, $R_{S}$. In other words, when the current secrecy capacity $C_{s}$ is not more than the pre-stablished threshold, the secrecy outage happens, which declares that the current secrecy rate cannot guarantee the security requirement [37,38]:

(14) $$P_{\mathrm{out}}\left(R_{S}\right)=\mathrm{Pr}\left\{C_{s}<R_{s}\right\}.$$

Intercept probability is defined as the probability that the transmission rate of the legitimate channel falls below the rate on the eavesdropper’s channel (secrecy rate becomes non-positive). It means that the wiretap channel has better SNR.

(15) $$P_{\mathrm{int}}=\mathrm{Pr}\{\gamma_{B}<\gamma_{E}\},\;$$

where $\gamma_{B}$ and $\gamma_{E}$ denotes the main (Alice) and eavesdropper SNR, respectively.

PLS issues are inherently connected with communications systems. NOMA as an emerging technology becomes the subject of research focused on PLS. In the case of communication, it must take into account the classical wireless scenarios. Depending on the assumptions on the system, number, and types of users, different levels of security can be achieved.

In Furqan [30], the author distinguishes between two types of intruders: external (non-NOMA user) and internal (NOMA user). Moreover, the intruder is considered as active and passive. An active intruder may disturb or hinder the radio channel estimation. The passive intruder only eavesdrops on the work in the radio channel. The NOMA system can therefore be considered in terms of safety assurance regarding external and internal intruders (or both). Analysis of designs and solutions provided by PLS, especially for external intruders, shows that they are similar to that which are employed in covert channels. The following solutions can be mentioned here like power allocation, beamforming, artificial noise, and jamming. These methods are effective methods in the case of an external intruder, and less effective in the case of an internal intruder.

Another interesting solution is [39], where users are divided into multi- and unicast users. Everyone gets some information, and only a few of them get additional information. This is primarily of value for increasing the channel capacity, as information is sent to two groups of users simultaneously. In this scenario, a unicast user is the near user and multicast users are far users (weak users). As with the previous case, the optimal solution to increase PLS is based on beam forming and power allocation [40].

Multicast transmission in MIMO systems and PLS issues were reflected in Xiao et al. [41]. It is assumed here, that the base station (BS) communicates with users of different security levels. The division exists due to the so-called “NOMA” and “non-NOMA” users. Generally, non-NOMA users do not perform SIC operations. The presented hierarchical model is based on a proper power allocation and the knowledge of path loss.

It can be inferred from the above literature that PLS in NOMA is investigated in different scenarios, such as SISO, MIMO, and large-scale networks. Particularly, solutions such as power allocation, transmit beamforming, transmit antenna selection, dividing users into groups, and generally via appropriate resource allocation, are provided to enhance the security of NOMA. The main problem in providing strong security is the SIC process in the case of an internal intruder. Generally, a SIC scheme in NOMA-based system does not guarantee secrecy [42]. If SIC can be successfully performed at the eavesdropper, it means that the information has already been compromised. Thus, there are many research opportunities in this area.

### 5.2. LPD

To guarantee security and confidentiality, it is often not enough to protect the content of the exchanged messages, i.e., in terms of PLS, and to hide the very fact of wireless transmission [43]. Concealment of wireless transmission may also be desired directly by state and military organizations (e.g., communication of aircraft with ground operators). Hiding the radio transmission is partially included in spectrum spreading techniques and is the only solution used today. However, the possibilities and limits of concealing transmissions by spreading have not been thoroughly investigated and analyzed. Thus, there are no qualitative parameters that would indicate the quality of the concealment of the distributed transmissions. Due to the lack of evidence and guaranteed results, the main reason to use spread spectrum signals is to ensure immunity to interference and obtain high reliability and data volume. Concealing information by distraction is, therefore, of secondary importance. The continuous need of transmitted data security has forced a new look at the spread spectrum under the new paradigm, known as low probability of detection (LPD). Increasing interest in LPD has been observed since 2013 [44]. Existing subject-related research focuses on three complementary groups of issues. The first group consists of the limits of the LPD (e.g., [45]). These works aim to determine the amount of information that can be transmitted with an overlooked probability of detection. The second group consists of the coding schemes and the characterization of the required key size to achieve LPD transmission (e.g., [46]). The third group focuses on the study and attempt to increase LPD connectivity in real conditions. The detection of covert channels is an issue in the field of steganography, or steganalysis [47,48].

Initially, steganography was considered mainly in terms of hiding information with a cover of a given size. There emerged a question of the amount of information that could be concealed when we have a given cover size and how to evaluate a given method of covert. For this purpose, initially, rates were provided, such as the number of bits sent per second, or bits sent per pixel (if the image is considered). However, these rates were often not compared with each other. Research began on how cover size affects detectability and security. Research on batch steganography, by concealing messages in many objects/covers, showed that N’s steganographic capacity for N various covers is equal to $O(\sqrt{N)}$ (square root law). Thus, the concept of steganographic capacity was introduced, which is understood differently than the capacity of the channel in a noisy environment (expressed by Shannon’s law). In Shannon’s law, channel capacity is only related to channel properties. By steganographic capacity, we mean the amount of information that can be embedded in the cover so as not to be detected. Another definition is the payload, which can be securely placed in the cover using a specific data embedding method. Therefore, the very definition of steganographic capacity is quite loose and not very precise. Steganographic capacity depends on the channel and the detection function. In Anderson [49] there is a statement that reads “Thanks to the Central Limit Theorem, the more cover text we give the warden, the better he will be able to estimate its statistics, and so the lower the rate at which [the steganographer] will be able to tweak bits safely. The rate might even tend to zero...”. The square root law is derived from the application of an energy detector (radiometer). In the case of a radio channel, by N we understand the so-called “channel use”, which defines the elementary portion of resources used in the system in time and frequency. Thus, this may, e.g., refer to the transmitted QAM symbol.

The square root law for the AWGN channel is presented by Bash et al. [43]. In the AWGN channel, we find additional variables that allow us to send more information. This is the noise variance on the guardian radio channel $\sigma_{w}^{2}$ and the noise variance on Bob’s channel $\sigma_{b}^{2}$, for which the square root law takes the form of $O\left(\frac{\sigma_{w}^{2}}{\sigma_{b}^{2}}\sqrt{N}\right)$. According to Lee et al. [50], it is possible to transfer O(N) bits of information, assuming that the intruder does not have full knowledge of the channel shared with Alice, which is associated with noise uncertainty. For this purpose, pseudo-random noise (artificial noise) can be used [51,52]. On the other hand, in Tandra et al. [53], the author proves that the detector will not find the signal if the SNR value at the receiver input drops below a specific noise threshold. The values presented above are theoretical, and the detector performance must be tested for each adopted scenario. Thus, another parameter/metric used in steganography is detectability. This is a measure of the probability that warden will detect a transmission between Alice and Bob, whether Alice is transmitting over a covert channel or not. So, it is the sum of the α + β probabilities, where α is the true positive probability, and β is true negative (covert channel detection when not present/Alice is not transmitting).

In general, covert communication is based on the existence of noise that the adversary (the warden) cannot accurately distinguish between the signal and noise. The performance of covert communication can be improved by increasing the measurement uncertainty of the adversary. Interference experienced by the warden is helpful for Alice [54]. In this way, she can achieve undetectable communication with better performance. Interference and jamming in a typical wireless communication that is focused on the high data rate is considered as harmful to efficiency. However, in the case of covert communication, it can be a useful security tool. Moreover, the covertness constraint requires that the output distribution, when the transmission takes place and the distribution when no communication takes place, must be almost indistinguishable. So, the potential solution is to modulate secret information into artificial noise that is distributed as the real channel noise. The discrepancy between the two distributions is usually measured by the Kullback–Leibler divergence.

Most of the current research is focused on detection based on energy detection. Detection means the verification of hypotheses. Thus, the way to describe the system when determining the null and alternative hypotheses is fundamental. In Shahzad et al. [55], the author generally refers to the creation of a covert channel, whereas the authors of [56,57] refer specifically to the NOMA system. However, there is a difference in the definition of the signal, which directly impacts the detection of the transmission by the radiometer. Assuming the transmitter’s power $P=P_{1}+P_{2}$, $x_{1}$—cover signal/weak_user, $x_{2}\;$—covert information/strong_user, in the case of [55,56], the signal is transmitted in the form of

(16) $$s\left(t\right)=\left\{\begin{array}{c} \sqrt{P_{1}}x_{1}\left(t\right)+n\left(t\right),\;for\;H_{0},\\ \sqrt{P_{1}}x_{1}\left(t\right)+\sqrt{P_{2}}x_{2}\left(t\right)+n\left(t\right),\;for\;H_{1}. \end{array}\right.$$

However, in the case of [57],

(17) $$s\left(t\right)=\left\{\begin{array}{c} \sqrt{P}x_{1}\left(t\right)+n\left(t\right),\;for\;H_{0},\\ \sqrt{P_{1}}x_{1}\left(t\right)+\sqrt{P_{2}}x_{2}\left(t\right)+n\left(t\right),\;for\;H_{1}. \end{array}\right.$$

It follows that the using a radiometer, ignoring all uncertainties and noise, in the first case (16), the measurement value is, respectively, $P_{1}$ and P (plus noise) for hypotheses $H_{0}$ and $H_{1}$. In the second case (17), the measured value will always be P (plus noise), and the energy detector will never detect the signal $x_{2}$. The situation will change when we introduce the concept of an informed user/receiver (or depending on the context—a NOMA user). If it is possible to perform the SIC (demapping) operation in the intruder’s receiver, the hypothesis (17) may be written in the form of

(18) $$s\left(t\right)=\left\{\begin{array}{c} n\left(t\right),\;\mathrm{for}\;H_{0},\\ \sqrt{P_{2}}x_{2}\left(t\right)+n\left(t\right),\;\mathrm{for}\;H_{1}. \end{array}\right.$$

In this case, the energy detector can identify the signal (the measurement result is noise or $P_{2}$+ noise).

NOMA is typically analyzed in the category of physical security (PLS), but there are works in the literature on the LPD topic. As an example, based on the model described by (17), the security aspect of the NOMA systems was considered in Ta [57]. The author examines whether the warden is able to detect the covert transmission between Alice and Bob, assuming that the covert channel is the strong user’s channel (in general, the strongest channel when there are many users in the system). The NOMA signal is always of the same power, regardless of the number of users. Hence, an external observer, equipped only with an energy detector, is not able to identify covert emission. The main conclusion, in this case, is that the detection of the covert signal is possible when the warden is able to perform the SIC operation for weaker users.

Similarly, as in the case of PLS, there are some metrics to evaluate LPD, but generally, they are less popular. In Chen et al. [16], the author notes that there is very little work on the detection of radio covert channels. Most of the studies deal with image steganography and network steganography in timing channels. In Dutta et al. [19], the author proposes the analysis of covert radio channels by examining the error vector magnitude (EVM), peak to average power ratio (PARP) or changes in the average signal strength. The following methods are derived from techniques used, for example, in statistic-based detection techniques. However, statistical methods require a sufficiently large amount of data (signal samples) to create a sufficiently accurate model and determine the detector thresholds. In Cao et al. [20], the author proposes the Kolmogorov–Smirnov test (KS) and the regularity test. The KS test compares the differences in the probability distribution. The regularity test is based on higher-order statistics. The proposed KS test defined for the empirical distribution functions of the cover $F_{1}\left(x\right)$ and the steganographic signal $F_{2}\left(x\right)$ is

(19) $$KSTEST=max(\left|F_{1}\left(x\right)-F_{2}\left(x\right)\right|.$$

The regularity test of [20] is a solution proposed for the timing channels [58]. In contrast to Cabuk et al. [58], instead of looking into a regularity test for one sequence of samples, two regularity tests are being tried for the cover and stego (covert), respectively, according to the following formula:

(20) $$regularity=STDEV\left(\frac{\left|\sigma_{i}-\sigma_{j}\right|}{\sigma_{i}},\;i<j,\;⩝i,j\right)$$

and then the absolute value of their difference is calculated as $\left|{\mathrm{reqularity}}_{cover}-{\mathrm{reqularity}}_{stego}\right|$. When the received value is greater than a certain value Δ, it is assumed that covert information is transmitted over the channel.

Having different detector solutions, methods of comparing their performance are needed. In Ker et al. [59], it is proposed to apply comparative methods:

(a) Calculation of the AUR where AUR = 0.5 corresponds to a random detector.
(b) Calculation of the minimum sum of the binary detector for false positive and negative errors, defined as $P_{E}=\frac{1}{2}\min\left(f_{p}+f_{n}\right)$ or $1-P_{E}$, where $f_{p}$ is false positive and $f_{n}$ is false negative.

In conclusion, it should be noted that recent scientific research indicates the potential possibilities of implementing LPD techniques in next-generation networks (5G and IoT) to protect privacy and confidentiality of data. Furthermore, concerning state security, it is important not only to decrypt messages sent (PLS) but also to detect radio emissions. Therefore, the issue of LPD belongs to the area of national security. Regulations covering the use of LPD information transmission should therefore be expected in the future.

## 6. Current Issues and Future Research Direction

Development of wireless communication, especially for 5G, is focused on NOMA systems. The issues related to the watermarking and covert channel are taken less into consideration. Most of the investigations connected with NOMA are focused on improving performance aspects. It seems to be obvious that future communication systems must be able to overcome a significant increase in capacity, data rate or energy consumption. NOMA gives the opportunity to effectively share resources, such as frequency and time. The main weakness of this solution is the security aspects. In the case of NOMA systems, SIC operations decode information from users that arrive collectively. For that reason, there is a security issue regarding user privacy and security problem. It is a big challenge to ensure that only legitimate users retrieve the desired information. The security problem in NOMA has its reflection in many publications and research [60,61,62,63].

Due to the broadcast nature of wireless communication, there is a high vulnerability to security attacks. There is a non-trivial trade-off between the data rate of the system and the security aspects. Moreover, most of the publications are based on theoretical analysis. In the future, the gap between theory and practice must be filled. Reinforcement of the NOMA security leads to the techniques typical for covert channels. These, in turn, lead to deteriorating data rates. It seems that, despite all the efforts to ensure security using physical dependencies, the answer to the question if cryptography is an unavoidable and complementary tool for sensitive data, is of great importance.

## 7. Conclusions

The article presents how, using one solution consisting of creating a radio waveform, which is a superposition of signals, we can watermark the transmission, create covert channels or multi-access systems. In this paper, we have illustrated some basic similarities and differences between them. The main purpose was to make it simple and understandable. A signal, in general, is generated in the same way, though the purpose of its use is distinct. All techniques are intended for a specific scenario. Watermarking is realized in a model with one sender and recipient. Covert channels can be realized in a model with one or two receivers (one for cover and another for covert information). The second one, as was shown, corresponds to the multiple access systems. Attention has been paid to the fact that NOMA, which is based on the signal’s superposition, gives additional summation channel throughput in comparison to OMA systems. Next, the two main approaches to security were shown, namely LPD and PLS. We have shown the main differences between LPD and PLS and presented the commonly used metrics to evaluate the secrecy performance of schemes. In this paper, we gave only a brief review of methods of improving transmission security. Especially for NOMA systems, due to their broadcast nature, they are vulnerable to eavesdropping. For this reason, realization of secure wireless transmission poses a challenge.

## Data Availability

The data presented in this study are available on request from the corresponding author. The data are not publicly available due to project restrictions.
